# Low-latency hierarchical routing of reconfigurable neuromorphic systems

**DOI:** 10.3389/fnins.2025.1493623

**Published:** 2025-02-04

**Authors:** Samalika Perera, Ying Xu, André van Schaik, Runchun Wang

**Affiliations:** International Centre for Neuromorphic Systems, The MARCS Institute for Brain, Behaviour and Development, Western Sydney University, Kingswood, NSW, Australia

**Keywords:** neuromorphic engineering, FPGA acceleration, multi-FPGA, networks on chip, transceivers, SNN, arbiter

## Abstract

A reconfigurable hardware accelerator implementation for spiking neural network (SNN) simulation using field-programmable gate arrays (FPGAs) is promising and attractive research because massive parallelism results in better execution speed. For large-scale SNN simulations, a large number of FPGAs are needed. However, inter-FPGA communication bottlenecks cause congestion, data losses, and latency inefficiencies. In this work, we employed a hierarchical tree-based interconnection architecture for multi-FPGAs. This architecture is scalable as new branches can be added to a tree, maintaining a constant local bandwidth. The tree-based approach contrasts with linear Network on Chip (NoC), where congestion can arise from numerous connections. We propose a routing architecture that introduces an arbiter mechanism by employing stochastic arbitration considering data level queues of First In, First Out (FIFO) buffers. This mechanism effectively reduces the bottleneck caused by FIFO congestion, resulting in improved overall latency. Results present measurement data collected for performance analysis of latency. We compared the performance of the design using our proposed stochastic routing scheme to a traditional round-robin architecture. The results demonstrate that the stochastic arbiters achieve lower worst-case latency and improved overall performance compared to the round-robin arbiters.

## 1 Introduction

Simulation of complex biological neural systems is a promising research area in the current era, as the brain performs computation in a power-efficient and impressive parallel manner that standard computers can't match. Though conventional von Neumann architecture-based designs offer shorter design times and configurability, larger-scale software-based simulators can't match biological spiking rates (ms) and demand significant power (Sharp et al., 2012). Consequently, researchers are increasingly exploring analog and digital hardware solutions like BrainScale (Pfeil et al., 2013). Application-Specific Integrated Circuits (ASIC) processors, such as TrueNorth from IBM (Cassidy et al., 2013), SpiNNaker (Furber et al., 2013), SpiNNaker 2 (Mayr et al., 2019), and the Loihi (Davies et al., 2018) chip and Loihi 2 (Intel Corporation, 2021), are being developed. FPGAs have also emerged as a promising area of study, providing significant speed-ups over software simulations (Javanshir et al., 2022; Schuman et al., 2017). Modern commercial neuromorphic processors, such as SynSense's Dynap-CNN, Speck, and Xylo, which are specialized for real-time vision processing, as well as Innatera's low-power mixed-signal spiking processors for audio and healthcare applications, are also emerging as promising solutions (SynSense, 2024; Richter et al., 2024; Bos and Muir, 2022; Innatera, 2024). Additionally, FPGAs offer the benefits of shorter design periods, reconfigurability, reusability for different applications, optimisation for each problem, and easy interfacing with host computers (Liu and Wang, 2009). Modern entry-level FPGAs contain many logic gates and physical memory, allowing large-scale neural networks to be created at a lower cost compared to other hardware options. Increasing the number of neurons not only demands more computing cores but also leads to inefficiencies due to excessive core-to-core communication, creating a bottleneck in scaling hardware for brain-scale SNNs (Fang et al., 2018). To address these challenges in large-scale SNN simulations, it is essential to interconnect multiple FPGA boards into a multi-FPGA Network on Chip (NoC) platform. Such platforms are crucial for providing high-performance solutions for computationally intensive tasks, logical emulation, and rapid prototyping, effectively overcoming the limitations of single-board configurations (Wang et al., 2015).

NoC switches are introduced between cores, offering a more scalable and flexible topology compared to traditional bus-based architectures. This multi-FPGA platform requires high bandwidth and low-latency communication to achieve real-time benefits. Good routing mechanism selection and exploration of topology between these nodes define overall design performance. Network routers are critical for directing data between senders and receivers, adhering to specific algorithms. They consist primarily of input buffers, crossbars, arbiters, and control units. An arbiter is key to managing data flow and resolving output port conflicts, ensuring smooth packet transfer through a network. Input buffers hold data temporarily, while crossbars connect ports under arbiter and control unit guidance (Kayarkar and Khurge, 2016).

Congestion in routing architecture significantly causes increased latency and data loss. Generally, there are two types of congestion that can occur. The first type occurs inside a router when multiple incoming packets request the same destination port. In this case, only one port is granted to transmit while other ports are stalled temporarily until transmission is complete. We refer to this as contention congestion. The second type is buffer congestion, which occurs when a destination router's input buffer is full; the router must wait until packets stored in the destination are transmitted. A packet will be dropped when buffer congestion occurs. Another factor that impacts performance is a router's buffer size. A larger buffer size can reduce the congestion rate. However, a dual-clock buffer is expensive in terms of area consumption.Therefore, in this work, we explain an arbitration strategy without utilizing a larger buffer size.

To address congestion and improve communication efficiency in NoC systems, various topologies have been proposed, each presenting distinct advantages and limitations. The mesh topology arranges routers in a grid, offering robust fault tolerance by providing alternative paths for rerouting data in the event of failures (Williams, 2023). However, this structure is often constrained by high congestion levels and increased latency, particularly for communication over longer distances (Hu et al., 2008). A refinement of this design is the torus topology, which links the edges of the grid to create a toroidal network. This enhancement reduces latency and improves scalability, but the additional interconnections significantly complicate design and lead to increased power consumption (Hu et al., 2008). Another commonly used topology is the tree structure, which supports scalability through its hierarchical organization. While effective for systems requiring structured communication, tree-based designs can suffer from latency issues due to the need for intermediate routing, especially under heavy traffic conditions (Kamal et al., 2012). The ring topology, by contrast, connects routers in a circular loop, achieving simplicity and fault tolerance through its ability to reroute traffic during failures (Williams, 2023). Nonetheless, it is often constrained by latency bottlenecks, as packets may have to traverse multiple nodes to reach their destination (Hu et al., 2008). While hierarchical tree-based topologies are widely recognized for their scalability and structured communication, they can encounter challenges with congestion at intermediate nodes, particularly under heavy traffic conditions (Kamal et al., 2012). To mitigate these challenges, we propose a routing arbiter mechanism specifically designed for hierarchical multi-FPGA systems. By incorporating stochastic arbitration, the mechanism adapts to traffic demands in real time, prioritizing data based on FIFO levels. This approach reduces congestion, improves buffer utilization, and enhances overall efficiency. By optimizing buffer performance and minimizing bottlenecks, the proposed mechanism provides a latency-efficient solution for hierarchical routing in multi-FPGA systems.

The proposed hierarchical tree-based routing architecture reduces the risk of deadlock by avoiding cyclic dependencies, which are common in ring or mesh topologies. The directed and acyclic structure ensures that data flows in a structured manner, minimizing circular waiting conditions. Additionally, the system employs buffer monitoring mechanisms to prevent overloading. When buffers approach capacity, traffic from upstream nodes is temporarily paused, helping to avoid congestion. Whilst the focus of our validation has been on general system stability, the architecture is designed to handle scenarios that could lead to deadlock, ensuring smooth operation under heavy traffic conditions.

Building upon the hierarchical tree-based routing architecture discussed earlier, this work proposes a novel routing arbiter mechanism for hierarchical multi-FPGA architecture that employs stochastic arbitration and considers the data levels of FIFOs, leading to more efficient buffer utilization and reducing the need for larger buffers. This mechanism effectively reduces the bottleneck caused by FIFO congestion, resulting in improved overall latency. We compare our stochastic arbitration technique to the standard round-robin arbitration technique to benchmark the latency performance. The proposed work presented in the paper discusses latency-wise, efficient hierarchical routing architecture for a multi-FPGA SNN hardware design.

The remainder of this paper is structured as follows: the next section provides an in-depth discussion of recent architectures of multi-FPGA SNN hardware designs within the context of related works. Following this, we delve into the details of our proposed architecture, explaining its components and experimental setup, and the corresponding results. Finally, we draw conclusions based on our findings, offering a comparative analysis and engaging in a comprehensive discussion of the implications of our work.

## 2 Related works

In the literature, various multi-FPGA architectures have been proposed for neuromorphic scalable SNN accelerators (Wang et al., 2018; Moore et al., 2012; Sripad et al., 2018; McDaid et al., 2008; Karim et al., 2020). Considering recent FPGA-based multi-FPGA SNN accelerators, the EMBRACE (McDaid et al., 2008) architecture features a router with a programmable address table, enabling the simulation of different SNN structures. The router is packet-switched and follows round-robin principles. It is a multi-FPGA custom system with a predicted capacity of up to 64 FPGA nodes, targeting high bandwidth and low latency communication. Modern FPGAs with integrated high-speed serial links were leveraged to simplify the construction of a cost-effective multi-FPGA system. The routing scheme used in Bluehive is a simple dimension-ordered routing scheme, a distributed deterministic routing scheme applied in n-dimensional meshes (Moore et al., 2012). This design approach enables the simulation of large networks, with 64k spiking neurons per FPGA and 64M synapses. Additionally, the design is scalable to a large numbers of FPGAs, and they have already demonstrated a four-FPGA system with 256k neurons and 256M synapses. Given the low utilization of inter-FPGA bandwidth, they predict linear scaling to at least 64 FPGAs, with a mean bandwidth of 250Mbit/s between each FPGA board. The limiting factor is FPGA-to-FPGA bandwidth. Based on a 3D torus configuration, the system achieves 12 Gbit/s of bidirectional bandwidth per channel. However, the design lacks a mechanism to address congestion within the SNN network during saturation scenarios, prioritizing a lightly loaded network to minimize congestion and emphasize low-latency routing. SNNs for Versatile Applications (SNAVA) is an FPGA-based platform for SNN hardware, as presented in Sripad et al. (2018). The primary motivation behind this work is scalability, with a proposed network topology based on a ring configuration. Unlike the approach shown by Moore et al. (2012), they avoided using extra hardware to connect numerous FPGAs, reducing the need for expensive dedicated interfaces to link multiple boards. They utilized pipeline operations on each board to increase communication performance.

Astrobyte, as detailed in Karim et al. (2020) and Karim (2020) is a multi-FPGA-based architecture developed for simulating spiking astrocyte neural networks (SANN). This work introduces a novel approach, combined with NoC, to facilitate dense communication between astrocytes and neurons. They have used conventional mesh routing topology with a round-robin arbitration technique. In Astrobyte, as detailed in Karim et al. (2020), they demonstrated that the platform could achieve up to ×188 speedup for SANN applications when compared to an equivalent MATLAB model. The Astrobyte architecture consists of bidirectional Intel Gigabit Transceiver Blocks (GXB) to serialize the data stream and send it over Serial Advanced Technology Attachment (SATA) connections.

The IBM Neural Computer Architecture (Narayanan et al., 2020) is a highly scalable parallel processing system with hundreds of programmable nodes in a 3D NoC mesh topology. The communication network of that design supports directed and broadcast packet routing schemes. Nodes are interconnected using single-span and multi-span Serializer-Deserializer (SERDES) (Sheldon, 2000) links connected to the fabric of the Xilinx Zynq FPGA. The system utilizes Postmaster Direct Memory Access (DMA) operation. An application running on the source node (either on the CPU or FPGA) writes data to a transmit queue on the FPGA logic. Upon reaching the destination node, the data is consumed by the FPGA and then written into a memory-mapped region. It is important to note that while the Postmaster DMA operation is a vital component, the broader IBM neural computer architecture is not limited to computational neuroscience; it also encompasses machine intelligence domains. IBM design's goal is more general than computational neuroscience. The IBM Neural Computer (INC) differs from others because it is composed of distinct processors + memory nodes interconnected by communicating links. Also, communication interfaces are not predefined and are not limited to a set of use cases like other systems. When we consider the above-mentioned multi-FPGA accelerator's communication architectures, they differ according to the focus of these designs. As an example, EMBRACE work is implemented to support programable different SNN structures (McDaid et al., 2008), Bluehive targets massive real-time simulations (Moore et al., 2012), and the work presented in McDaid et al. (2008) focuses on SANN acceleration and a runtime data acquisition system. However, router architecture design, arbitration technique, and topology used in these designs vary based on each focus. The main distinction of our work is that we aim to achieve a latency-efficient architecture. In the SNN simulations, since spike arrival time encodes the spike data, a minimum latency requirement provides an advantage for accurate communication.

## 3 Architecture

**Figure 1:**
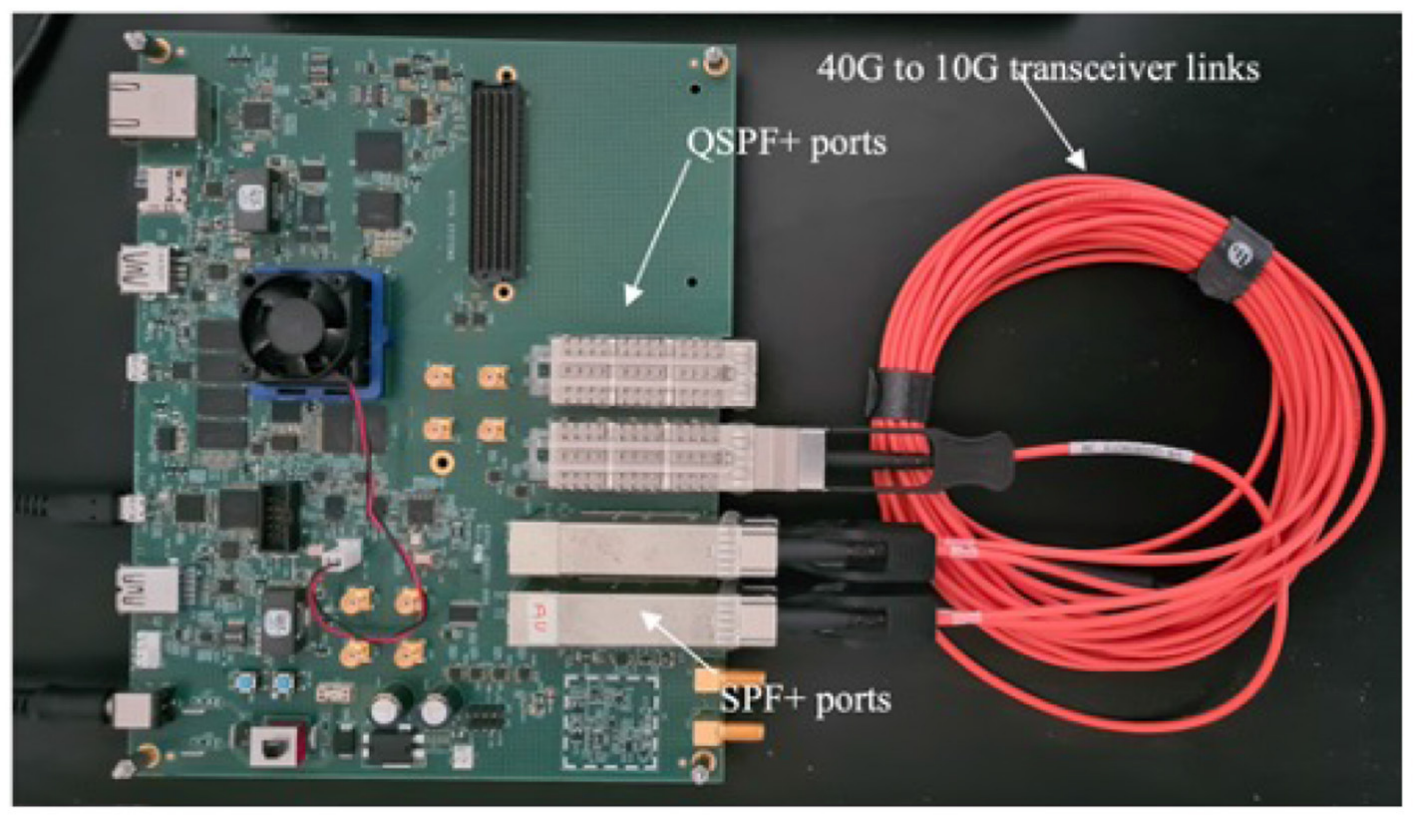
Arria 10 SoC development FPGA board, where the orange color cable is 3m Generic Compatible 40G QSFP+ to 4x10G SFP+ Breakout Active Optical Cable.

We use a NoC hierarchical tree-based topology in our design. In computer networks, tree topology is sometimes known as a star bus topology (Williams, 2023). This nomenclature arises from the fusion of features inherent to both star and bus topologies, culminating in a tree-like structure. The proposed multi-FPGA architecture spans two main hierarchical levels, reaching 128 nodes at the bottom level (L1) of the hierarchy, with level two (L2) routers communicating with each other. We use an extended version of the hierarchical address-event routing (HiAER) architecture (Park et al., 2017) for scalable communication of neural and synaptic spike events between different cores. The HiAER adopts a tree-based interconnect with a fractal structure for its connectivity hierarchy. Interestingly, the communication bandwidth remains consistent at each level. This consistency is attributed to the decreasing fan-out observed at higher levels of the hierarchy. Therefore, additional nodes can be added to the design easily by expanding hierarchical levels while maintaining communication bandwidth at each node. The proposed architecture implements routers and computing nodes on FPGA and high-speed transceiver links are used as inter-node communication links. Data packets are used to organize and transmit information between FPGAs. A packet typically consists of the actual data payload that needs to be transmitted, along with additional control information such as headers and source/destination addresses. We implemented the functional verification of our prototype system on different FPGA boards, which we discuss in the experimentation and results section. In this setup, with four FPGAs working together, we have explored approaches for different levels of communication. Initially, we calculated performance evaluation matrix data with a four-FPGA system. Subsequently, we compared this data with an emulated loopback prototype system. More details on data collection experimental setups for this four-FPGA system and the loopback prototype system are in the experimentation and results section. For clarity, we will refer to the four-FPGA setup as the “Non-Loopback Setup.” From this point onward, any mention of this setup will be under this name. Conversely, the loopback prototype system on the Arria 10 SoC FPGA board, depicted in Figure 1, will be referred to as the “Loopback Setup.”

The Arria 10 SoC FPGA is specifically designed to develop high-performance networking applications. Its high-performance FPGA fabric allows complex designs and a wide range of high-speed interfaces, including transceiver links (Hitek Systems LLC, 2021). The board consists of four SFP+ (small form-factor pluggable ports) for 10G/1G interfaces and dual QSFP+ (quad small form-factor pluggable ports for 40G interfaces (CablesAndKits, 2023). The prototype functional verification design has been implemented on this FPGA by loopback in transceivers links into the same SoC FPGA, as shown in Figure 1. We have used 10G transceiver link interconnects in the design. Our choice of high-speed transceiver links for our multi-FPGA setup hinges on their fast data rates, low latency, extended reach, and high reliability. These links can move data at gigabit speeds, crucial for the high-volume data transfers needed between FPGAs. They also offer low-latency data transmission, key for efficient inter-FPGA communication, and include error-handling features for dependable data flow (Stackler et al., 2018; Venkata et al., 2013). To clarify the system's timing approach, the proposed design operates as a locally synchronous architecture with a single, shared clock domain. All components in the system are synchronized to this clock, ensuring consistent timing across the design. The system operates at a frequency of 100 MHz.

### 3.1 Router structure

**Figure 2:**
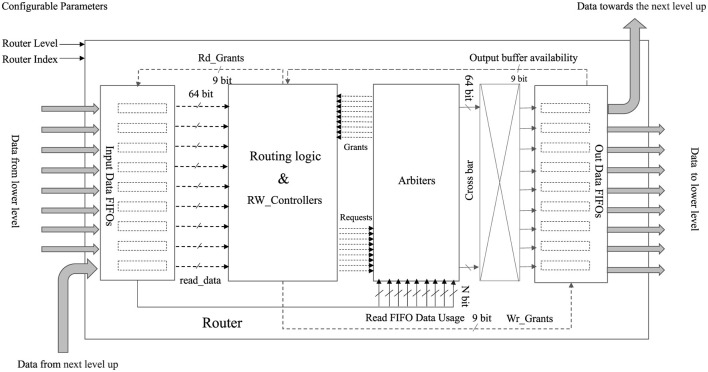
Simplified block diagram of the router: this diagram illustrates the architecture of the router, showcasing nine input FIFOs and nine output FIFOs responsible for managing 18 input-output ports, ensuring smooth data transmission. The RW_Controllers block facilitates data reading from the input FIFOs and exerts control over data writing toward the specified output direction. In every clock cycle, the Arbiter utilizes the input data usages from the read FIFOs to make decisions regarding granting requests.

The block diagram of the router structure is shown in Figure 2. As shown in Figure 2, each router handles nine inputs and nine outputs, forming eighteen input-output ports for data transmission. The key advantage of the router is that it can be configured for any level of hierarchy in the system by specifying the level and index of the router as a configurable parameter. This configuration flexibility allows the same router to be used at multiple levels of the hierarchy, eliminating the need to create multiple routing models for different levels of the system. Therefore, a configurable router provides greater flexibility and scalability in the design of a system, allowing for easier modifications and adjustments to changing design scalability requirements. It is important to note that in this context, changes necessitate recompilation of the design, as it does not support runtime reconfigurability. The router consists of eight input ports designed to receive data from a lower level, forming a configuration resembling a star cluster. Moreover, one input port is exclusively designated for communication with the upper or higher-level router. Employing a store-and- forward flow control mechanism, the router stores incoming data within nine FIFOs, subsequently forwarding this data based on the corresponding control mechanism. Specific threshold levels are defined for each input FIFO to ensure data integrity. For instance, if the FIFO width is set at 1,024 (1k), the threshold is established at 1,012 to ensure there is enough room to accommodate another packet, as each packet contains twelve flits. Once this threshold is reached, a signal is triggered to indicate that the FIFO is reaching its capacity. This signal warns the transceiver's RX FIFO, prompting it not to read out data to prevent the loss of incoming data. Importantly, data is only written to the input FIFOs if they are not already nearly full.

The incoming data's format is referred to as “flits,” as described in more detail under the Data Transfer Format section. These flits possess a width of 64 bits, aligning with the chosen 64-bit width of the input FIFOs. The read FIFOs function under an Asynchronous First Word Fall Through condition, ensuring that data is available at the output port before the read request signal is asserted. Consequently, both the routing logic and the RW_Controllers block receive the subsequent read-ready data from the input FIFOs.

The routing logic component in the block diagram presents the responsibility of hierarchical tree-based network routing or hierarchical addressing. In hierarchical routing, the address space is divided into a hierarchical structure, with each level of the hierarchy denoting a distinct level within the network topology. When a packet flit undergoes routing via the hierarchical approach, the decision on where to route it depends on the hierarchical address of the target node. The routing algorithm first checks whether the destination node is in the same cluster or subnetwork as the current node. If it is, the packet is directly forwarded to the destination node. If not, the packet is forwarded to the next level of the hierarchy, either higher or lower, depending on the address of the destination node. Based on the routing logic decisions, each input FIFO-related routing logic block keeps sending the requests to the arbiters. There are nine arbiters for the output and nine port directions to grant requests for multiple access to the relevant side. Based on that, the RW_Controllers receive the grant signals and decide which FIFOs' read requests should be asserted to avoid conflicts. Moreover, RW_Controller is also responsible for writing requests asserted for output FIFOs based on the read data asserted and which direction output FIFO should be asserted.

Each input FIFO is linked to a separate routing logic and RW_Controllers block from the other input FIFOs. During each clock cycle, the stochastic process takes into account the number of words stored in each input FIFO. This consideration aids in the selection of a priority port. In essence, every clock cycle involves the shuffling and comparison of the number of words in each FIFO. In cases where all FIFOs contain an equal number of words, a random selection process determines which one gains priority for sending data in a specific direction. This comparison-based procedure leads to a scenario in which mostly filled FIFOs are more likely to be granted an opportunity to read out and write in a particular direction during each clock cycle.

The Arbiter efficiently manages the simultaneous granting of selection requests for output port directions, effectively avoiding conflicts that may arise from multiple write accesses to the same output direction. The arbitration and control mechanism's function is detailed under the Arbiter sub-section.

### 3.2 Data transfer format

In our custom protocol designed for use in a neuromorphic system, spikes are transmitted between neurons and synapses using an efficient AER-based NoC packet transmission method. The protocol transforms AER data messages into 64-bit width units, known as flits, which are compatible with the parallel inputs/outputs of the physical layer (PHY) transceiver link. The number of flits that comprise a message depends on total data capacity of the message itself. For instance, a message containing 512 bits of event data, along with an additional 100 bits for header information, would be broken down into one header flit and nine tail flits for transmission. The header flit, which contains the source and destination address, is transmitted first, followed by the tail flits, resulting in a total of ten flits for a 612-bit message. The position and sequence of these flits are indicated by Tail bits/Control bits in the four least significant bits of each flit. The control bit “0000” signifies a header flit, and subsequent control bits from “0001” to “1001” represent the sequence of the tail flits, ensuring the correct encoding and ordering of the message data.

### 3.3 Routing logic

Routing logic is the heart of the routing function and is responsible for hierarchical tree-based network routing or hierarchical addressing. The initial seven bits of the header flit represent the data read from FIFO_read_data [63:57], with these bits indicating the destination address within the hierarchy of 128 nodes. Consequently, the role of the routing logic block encompasses decoding the destination address from the header flit and initiating a request to the designated output port.

**Figure 3:**
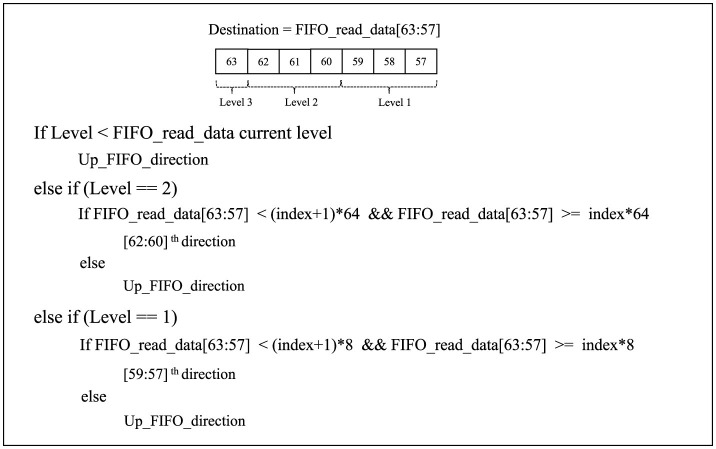
Routing logic pseudocode. Here, “Level” is the signal received for each router; for instance, it can be 1, 2, or 3, corresponding to the respective level, and the “index” is the router's index in each level of the hierarchy. For level 1, this is 0 to 7; for level 2, this is 0 to 1. Once the destination node's level range is determined based on [59:57], [62:60], and the 63rd bit of FIFO_read_data, respectively, for level 1, level 2, and level 3, the destination index is further determined.

The “level” and “index,” shown in red in Figure 2, are configurable parameters that allow the same router to be used at multiple levels of the hierarchy. Every FIFO reads data directed to the routing logic block to determine its output port direction. The pseudocode used in the routing logic block is shown in Figure 3. Based on the seven destination bits in FIFO_read_data [63:57], the first three least significant bits can be taken as the Level 1 index number. The subsequent three bits represent the Level 2 index number, while the final most significant bit represents Level 3. If a specific router level matches the readout data level, one should first verify if the node direction belongs to the same router cluster. If so, the output direction is chosen based on the FIFO_read_data [59:57] bits.

### 3.4 Arbiter structure

This work proposes a novel arbiter technique to address the latency issue caused by the contention congestion and improve the design's latency performance. The Arbiter receives nine sets of FIFO Usage Data from all nine input FIFOs and a nine-bit Request signal, which indicates which FIFO is requesting access in the Arbiter-assigned direction. The Arbiter stochastically compares the FIFO Usage Data and identifies the highest priority channel, referred to as the “Priority Channel.” If all the FIFO Usage Data are the same, and the Request is high for a specific direction, the Arbiter stochastically selects which one should be designated as the “Priority Channel” during that clock cycle. In this manner, the Arbiter continuously monitors data within the input-side FIFO queues using these signals and grants write requests to the appropriate output ports based on the number of words available in each FIFO that is requesting access. Furthermore, the Arbiter takes into account the availability of buffer space in a specific direction during each clock cycle when making a decision.

**Figure 4:**
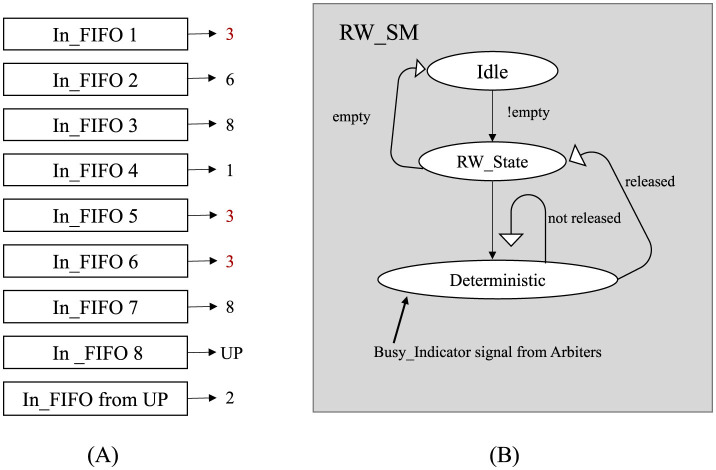
Conflict control state machine diagram for handling conflicts when multiple inputs access a single output direction. **(A)** illustrates a scenario where conflicts arise when multiple input FIFOs attempt to send packets to the same output direction. Each “In_FIFO” represents a queue holding packets waiting to be routed. The numbers on the right-hand side of each FIFO indicate the intended output direction of the packet. The red numbers highlight cases where multiple FIFOs are targeting the same direction, resulting in contention. **(B)** provides a simplified overview of the RW_SM state machine. The RW_State determines the input FIFO read operation based on the “released” signal from a specific output and a write grant for reading data in that direction. The “Busy_Indicator” signal is a global signal within a single router node that continuously monitors the write bus.

A critical component of this system is the Read Write State Machine (RW_SM), as depicted in Figure 4. Specifically, it manages read request grants from input FIFOs and write request grants for the desired output ports. The objective is to attain conflict-free access for every port. Within the context of a nine-port router, there exist nine RW state machines denoted as RW_SM_0 through RW_SM_8, where the number corresponds to the respective input port.

Each RW_SM relies on a signal called the “Busy_Indicator,” which monitors the write-busy state of output FIFOs. By utilizing the Busy_Indicator and Priority Channel signals, which represent grant signals from Arbiters, the RW_SM determines whether to activate the write signal for the target output channel of the currently requested data. While the RW state machine is in the read state, granting a Write request for the desired output direction requires satisfying at least one of two conditions. First, the input port number must match the input port number of the currently selected Priority Channel. Second, the requested output direction must not be busy, as signaled by the Busy_Indicator. This signal continuously monitors for conflicts when accessing the same direction. The RW_SM facilitates the transfer of read data to the intended output direction through the crossbar by utilizing the asserted write signal. Simultaneously, another read request can be granted. If neither condition is met, both the read and write request signals continue to persist until one of the conditions becomes true. This approach minimizes data loss and eliminates the need for intermediate buffer switches. Efficiency is improved by deferring the reading of new data until the current data are successfully written in the intended direction.

Simultaneous operation of all RW state machines leads to parallel processing, allowing for the simultaneous reading of multiple input channels and writing to the maximum feasible number of grants in each clock cycle. Additionally, prioritizing the most densely-populated input FIFOs (those containing the maximum number of words) during each clock cycle effectively contributes to alleviating buffer congestion.

Our arbitration process simultaneously supports many inputs and output channel read-write grants by computing buffer-used indexes. Supporting multiple-read request grants to avoid conflicts is not a novel concept. Still, the novelty of our arbiter mechanism is that the arbitration process involves the router FIFO's data levels for the multiple-read grants using a stochastic process. This simultaneous multi-output channel request grant causes a decrease in latency and throughput performance because considering the buffer utilization levels reduces congestion in the buffers.

### 3.5 Computational core: function and interface

In our design, the routing architecture is intended to support the implementation of SNNs. For testing and validation purposes, we utilize dummy processors to inject traffic into the system, simulating the behavior of neural networks.

Within this context, when a neuron receives sufficient input from its incoming signals within a short period of time, the membrane potential crosses a threshold, which initiates an electrical signal called an action potential. An action potential is also referred to as an “event” or a “spike.” The Computational cores in the design are responsible for generating event data based on the firing rates and engine connection parameters. We employ a Leaky Integrate-and-Fire (LIF) (Brunel and Rossum, 2007; Lu and Xu, 2022) neuron model in the computing nodes or dummy processors to generate event data. The dummy processors, adapted from the work presented in Wang et al. (2018), are used to inject traffic into the proposed routing architecture, to verify the architecture's correct operation. Each dummy neural processor consists of a total of 512,000 (512k) LIF neurons, distributed across its neural engines using a Time-Multiplexed (TM) approach. These engines generate stimuli by adjusting firing rate parameters. Additionally, by employing dummy processors instead of complete processors, we can optimize resource utilization to test processing nodes' inter-communication for a scalable system emulation. This optimisation enables the implementation of more dummy processor nodes in the loopback prototype system on the same FPGA board, effectively illustrating a scalable system. In future work, the dummy processors will be replaced by actual neural processor nodes.

**Figure 5:**
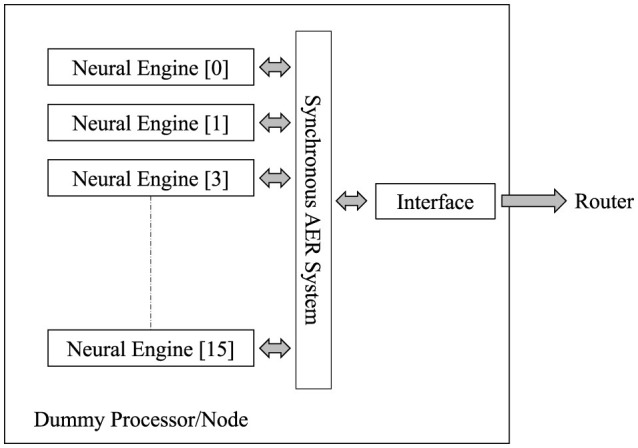
Overview of the dummy processor in the computation core. Sixteen neural engines are arranged in parallel within the system to enhance the processing capacity. Each dummy processor contains an interface module responsible for converting AER data into random event packets.

The LIF network modeling integrates input currents over time and generates a spike when the membrane potential reaches a threshold. After spiking, the membrane potential is reset to a resting state. The dummy processor overview is shown in Figure 5. There are 16 neural engines connected in parallel in the system. Each neural computing core receives incoming address event data and generates outgoing address event data. Linear Feedback Shift Registers (LFSRs) are used to generate a pseudo-random sequence of bits, which compare with the firing rate configurable parameter given to each processor to generate the events. This allows the firing rate parameter to control the probability of spike generation per time step.

This Computational core module consists of an interface module for each dummy processor, which generates random event packets. Each event message data has a header (101 bits) and 512-bit event data. These data flits are transferred to the Router to send it to the corresponding destination. The bits [67:64] of the header define the index of the destination FPGA board. We use only one header when transmitting data to simplify the data structure and minimize overhead. By examining bits [67:64], we determine the destination of the incoming message data. Subsequently, we modify the message to align with the data transfer format, ensuring it includes just one header. The different experimental setups measure the performance of the design with different traffic forward directions.

To demonstrate our concept, we utilized the Arria 10 FPGA board. The Arria 10 SoC FPGA is designed explicitly for high-performance networking applications and supports a broad range of high-speed interfaces, including transceiver links (Hitek Systems LLC, 2021). This makes the Arria 10 particularly suitable for prototype development and concept testing, as it can efficiently handle the demanding requirements of these early-stage applications. However, the proposed concept will be used to scale up and experiment with large-scale neuromorphic supercomputer, called DeepSouth, which is built to enable the emulation of large networks of spiking neurons to simulate processing in the human brain (DeepSouth, 2024). DeepSouth hosts 92 Bittware cards with an Intel Stratix 10 FPGA each. The FPGAs are configured to emulate spiking neural networks in parallel (DeepSouth, 2024).

## 4 Experimentations and results

### 4.1 Experimental setup

The PC used for all the experiments in this work had the following specifications: a MacBook Pro with a 2.7 GHz Quad-Core Intel Core i7 processor and 16GB of RAM. Intel Quartus Prime 18.0 SE was utilized for FPGA design, synthesis, and programming. The Intel Quartus Prime software was installed on a Linux-based operating system that ran as a virtual machine on the PC. SignalTap was used for design verification and data collection.

A prototype design was implemented to demonstrate the proof of concept for a scalable architecture using four FPGA boards. This setup employs two FPGAs from the Arria 10 device family: 10AS066 and 10AX115. Additionally, the design incorporates two Terasic DE5-NET boards, each featuring the Intel Stratix V 5SGX EA7N2F45C2 model. For each of the lower-end FPGAs, the Terasic DE5-NET board is equipped with four 10G transceiver links. Consequently, we can implement four processors on one board. Using two boards we can accommodate eight processors in the Level 1 (L1) cluster. This is why we selected two Terasic DE5-NET boards for the prototype design. For the next level, specifically Level 2 (L2), our requirement was an FPGA board specifically designed for high-performance networking applications. It should possess ten 10G high-speed interfaces. This includes transceiver links: eight to connect to the lower dummy processors and one to communicate with the upper-level router, thus establishing a single cluster prototype design. Therefore, we chose the Arria 10 SoC FPGA for Level 2, which allows us to utilize 12 10G transceiver links on a single board. Furthermore, for our final design in the future, we plan to use the Arria 10 SoC board for Level 3 as well. When dealing with 128 nodes, one L2 board consists of eight L1 routers and should be able to communicate with neighboring L1 boards; therefore, it requires nine links per board. However, for the current prototype with eight dummy designs, we used the Arria 10-GX board for the Level 2 hierarchy since it only requires a single transceiver interface for one L1 cluster. The 10G High-speed transceiver optical fiber cables facilitate communications between the implementation nodes and routers, as well as inter-router communications. High-speed transceiver optical fiber cables are used for the prototype implementation for both node-to-router and inter-router communications.

**Figure 6:**
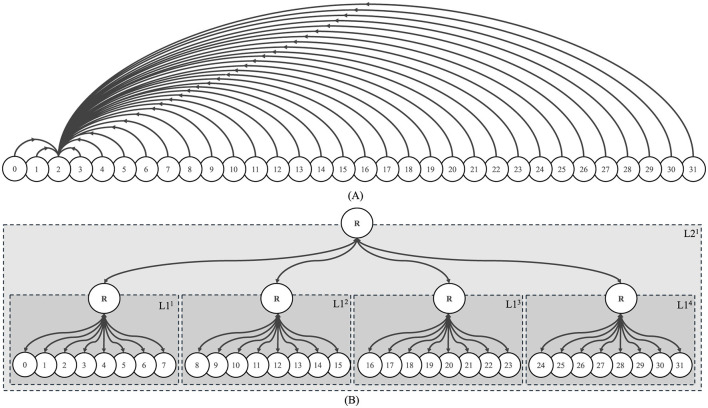
Experimental setup. **(A)** High-Level Traffic Flow: Each dummy processor sends traffic data to node two as its destination. Arrowheads represent the flow of traffic between nodes. **(B)** Hierarchical Representation of Routing Topology: A circle marked with an “R” signifies an L1 or L2 router, and each L1 cluster comprises eight dummy processors. Numbered circles in the L1 cluster represent processor nodes. Consistent node labeling and clear arrows are used to improve clarity. Note that nodes are numbered sequentially within each L1 router, starting from node 0 in the first L1 router and progressing to subsequent nodes in the next routers. The “R” circles indicate routers at different levels of the hierarchy.

The proposed hierarchical architecture is designed to support one-to-many and many-to-one communication. Fan-in (all nodes to one node) is achieved by aggregating data through hierarchical levels, where each L1 router collects packets from its connected nodes and forwards them to its parent L2 router, ensuring delivery to the target node. Similarly, fan-out (one node to all nodes) is inherently facilitated by leveraging intermediate routers for packet replication and forwarding. For instance, in the 32-node system illustrated in Figure 6, the hierarchical levels enable efficient communication. Each L1 router handles eight connected nodes, and the L2 router connects the four L1 routers. In a fan-in scenario, all nodes can communicate with a single target node by routing packets through their respective L1 routers, which aggregate the data and forward it to the L2 router. In a fan-out scenario, a single source node can send data to all 32 nodes by replicating packets at the L2 router, which forwards them to the appropriate L1 routers for distribution to the connected nodes. For example, in a 32-node system, the fan-out is 32, allowing a single node to send data to all other nodes. Similarly, in a 128-node configuration, the fan-out scales to 128, enabling communication with all nodes in the system.

### 4.2 Latency analysis

**Figure 7:**
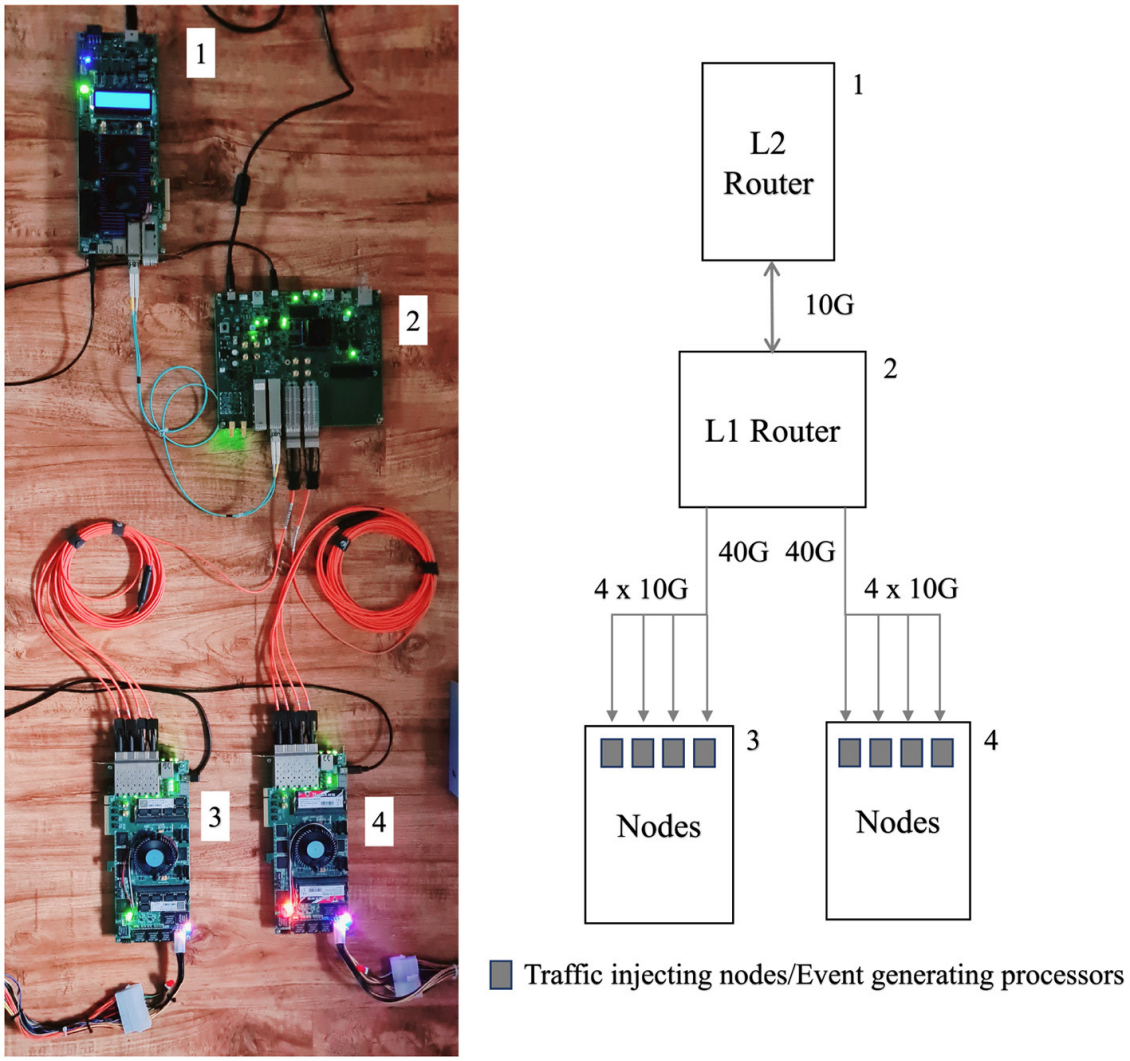
The system setup. In the diagram, Board 1 is Arria 10 GX, Board 2 is Arria 10 SoC and lower Board 3 and Board 4 are StratixV FPGAs. 10G and 40G into 4x10G breakout cables are respectively in blue and orange colors.

The prototype design is depicted in Figure 7. The right side of Figure 7 illustrates the block diagram of the prototype design, which is presented in a hierarchical architecture as depicted on the left side. The three hierarchical levels are illustrated in the prototype system where one level one router and one level two router and the lowest level eight traffic injecting nodes are implemented. Board 1 and Board 2 communicate with 10 G fiber optic cable, and lowest level Board 3 and 4 communicate with Board 2 using two 40G to 4x10G breakouts optical fiber cables. Both Arria 10 GX and SoC devices support a chip-to-chip data rate ranging from 1.0 to 17.4 Gbps per lane in different configurations. For the purposes of this work, all FPGAs utilize the 10.3125 Gbps data rate configuration.

In our research, we implemented an experimental setup for data collection, referred to simply as “Experimental Setup.” This setup features 32 dummy processor nodes which indicate the direction of traffic injection. While the initial representation may suggest a 32-node system, it is important to note that the actual experiments were conducted using a variety of node systems, all configured in the same manner as the described “Experimental Setup” in Figure 6. The intricacies and outcomes of this singular setup form the basis of our experimental analysis.

### 4.3 The PHY latency emulation

**Figure 8:**
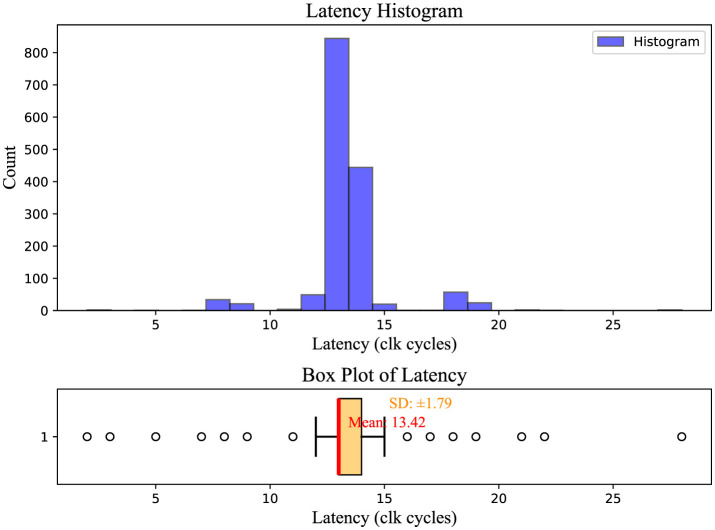
Latency histogram of PHY emulation. The *X*-axis shows latency clock cycles, and the *Y*-axis shows bin count per *Y*-axis value. The accompanying box plot below illustrates the distribution of latency values, highlighting the median (red line), interquartile range (light orange box), and potential outliers (black circles).

As a proof of concept, we implemented an alternative setup to test the proposed architecture with a different number of nodes on the same Arria 10 SoC FPGA board. We measured the transceiver loopback experimental latency using the setup illustrated in Figure 1. In this loopback system design, the inter-node interconnects also communicate via 10G transceiver links. The latency was calculated by measuring the number of cycles transmit data took to reach the receiver side of the transceiver at its loopback mode. The simulations were executed over 131,072 cycles, representing the maximum capacity for SignalTap. All the transmit data was captured at both the TX and RX sides. Latency was determined by exporting this captured SignalTap data. The average latency of all packets processed by the router is depicted in the histogram presented in Figure 8.

The average latency was 13.42 clock cycles, with the reference clock frequency of SignalTap set at 100 MHz. Therefore, the average latency was 134.2 n seconds.

The experimental setup is based on Experimental Setup shown in Figure 6. To calculate latency, we monitored the number of cycles required for packets to travel from the source node to the destination node. This process involved counting the clock cycles until the data reached its destination. To facilitate this calculation, we introduced a counter block, incrementing its value until the expected data reached the destination. Once the precise data was received, the next sending value was latched, the counter value was reset, and the process continued.

**Figure 9:**
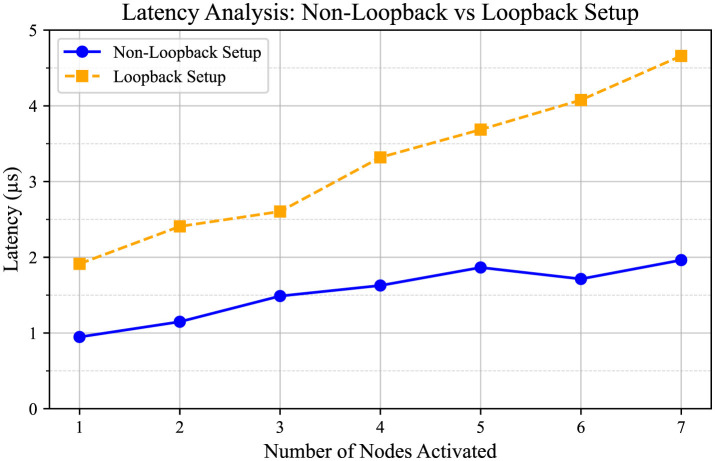
Comparison between Non-Loopback System and Loopback System. The Non-Loopback system is the design setup used in Figure 7, and the Loopback system is shown in Figure 1. Both setups used eight nodes. Node 0 initiates traffic injection at a 100% traffic ratio when other nodes are silent. Subsequently, the remaining nodes sequentially initiate traffic injection at the same 100% ratio.

Latency analysis was conducted for the four FPGA setup, referred to as the Non-Loopback setup and the Loopback setup, as shown in Figure 9. In the figure, the blue line represents the Non-Loopback System, and the yellow dashed line represents the Loopback System. The setup is similar to the experimental setup shown in Figure 6, with the main difference being that it employs up to eight dummy processor nodes. In this scenario, Node 0 initiates traffic injection at its maximum traffic ratio of 100%. Subsequently, other nodes begin injecting traffic at the same 100% ratio one after another, and the average latency is calculated. In the Non-Loopback Setup, the 0th processor node on FPGA Board 3, as depicted in Figure 7, sends traffic to the third node at its maximum capacity, while all other nodes on both Board 3 and Board 4 sequentially begin sending traffic to the system at 100% capacity.

Both setups utilized eight nodes. In both systems, Node 0 initiates traffic injection at a 100% traffic ratio when the other nodes are silent. Subsequently, the remaining nodes sequentially initiate traffic injection at the same 100% ratio. The *X*-axis represents the number of nodes initiating traffic at a 100% traffic injection ratio, while the *Y*-axis represents the average latency in seconds. Noticeably, the latency exhibits an increasing pattern as the number of nodes increases. Both plots exhibit similar characteristics, with latency values ranging approximately from 0.9 × 10^−6^ to 2.9 × 10^−6^ s. The results display latency for the case when the system operates at 100 MHz.

The comparison between Non-Loopback and Loopback systems, which were implemented on the Arria 10 SoC board, reveals that the latency of the Loopback system is slightly greater than that of the Non-Loopback system. This difference is reasonable, considering that in the FPGA setup, each node has its dedicated duplex transceiver link for transmitting and receiving data without sharing. In contrast, the loopback system involves shared utilization of the same link among multiple nodes. Consequently, in the loopback system, each data set experiences a certain amount of waiting time before it can be transmitted or received due to the shared nature of the link. Despite the slight difference in latency, the actual latency can be predicted based on the analysis conducted.

**Figure 10:**
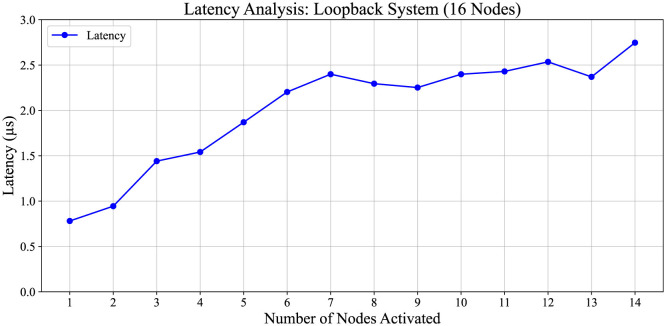
Latency analysis of a 16-node system. The experimental setup for latency analysis and traffic injection direction is similar to the setup depicted in Figure 6. In this scenario, Node 0 initiates traffic injection at a maximum traffic ratio of 100%. Subsequently, other nodes sequentially inject traffic at the same 100% ratio. Latency trends in a loopback system as nodes are sequentially activated. The results show that latency increases incrementally with up to seven active nodes. Beyond this point, latency transitions into a stabilization phase, likely due to the system's ability to efficiently distribute load and reach steady-state performance.

Figure 10 presents the latency analysis for the 16-node system, where each node initiates traffic injection at a 100% rate, one by one. In the plot, nodes 1 through 15 sequentially commence packet data injection into the network in the direction of Node 2. Subsequently, the average latency is computed for each traffic scenario. These experiments are performed using the experimental setup depicted in Figure 6 and are conducted on the Loopback Setup.

Figure 10 illustrates a latency variation pattern among 16 nodes, where traffic injection begins at a 100% rate. Latency significantly increases up to the seventh node and subsequently stabilizes at ~2.25 × 10^−6^ s.

**Figure 11:**
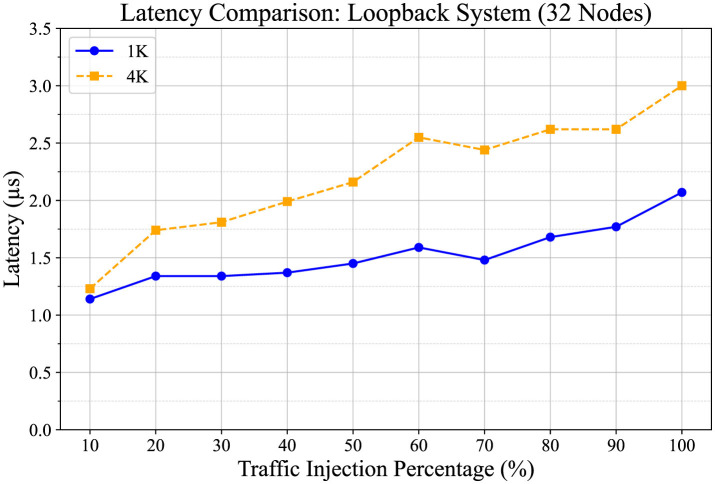
Latency comparison between designs with 1k and 4k input FIFO lengths, as a function of traffic injection percentage across all nodes (from 10% to 100%). The blue plot represents the latency of the design with 1k input FIFOs, while the orange dash plot represents the latency of the design with a 4k input FIFO system. The results indicate that the 4k FIFO design achieves slightly lower latency at most traffic injection rates, suggesting an incremental advantage in handling network loads.

The latency corresponding to two FIFO depth configuration scenarios for a 32-node system is depicted in Figure 11. We have used the setup of Figure 6 for this measurement with all nodes injecting traffic data into the system at incremental percentages of 10%, 20%, etc. The blue line in the graphs represents latency measurements as they vary with different traffic ratios for the routing architecture utilizing 1,023 (1k) data flow FIFOs in the router. Conversely, the orange line illustrates latency variation with different traffic ratios for the routing architecture employing 4,096 (4k) data flow FIFOs in the router.

In both scenarios, latency increases as the traffic injection ratio rises. The average maximum latencies recorded are 2.07 × 10^−6^ s for the system utilizing 1k FIFOs, and 3 × 10^−6^ s for the system using 4k FIFOs. We can define these as the worst case of latency since in these experimental setups, we identified the worst-case scenario as all nodes operating at their maximum traffic injection rate (100%), fully utilizing the system's bandwidth. This condition represents the highest load the system can handle and provides a reference for evaluating its performance limits.Furthermore, there is no significant latency variation between these two scenarios respectively.

**Figure 12:**
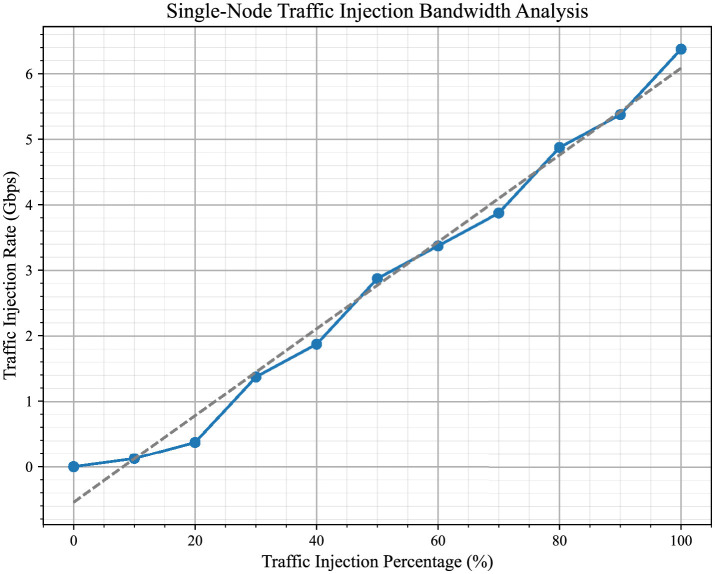
This figure shows how bandwidth changes as the traffic injection percentage increases from 10% to 100% for a single node. It give insights how the system performs under different traffic conditions, from average to maximum load.The linear trend aligns with the theoretical maximum bandwidth of the system, confirming its efficiency under full load conditions when operating at 100 MHz.

In this context, we have further analyzed the bandwidth variation as the traffic injection percentage increases from 10 to 100% for a single node. This is presented in Figure 12, which highlights how the system's bandwidth utilization evolves under varying traffic conditions. By illustrating the bandwidth trends, the figure provides insights into both average and high-load conditions.

When all processor nodes simultaneously inject traffic at their maximum capacity (100%) toward a single destination, we consider the system to be operating under its maximum load condition for that direction. This situation represents the highest load the system can handle and defines its performance limits. This scenario, referred to as the worst-case scenario, aligns with the experimental setup as shown in Figure 6, where the full system bandwidth is utilized for traffic directed into a single destination.

### 4.4 Round robin vs. stochastic

The proposed architecture, featuring a stochastic arbiter, is compared with an equivalent hardware setup that utilizes round-robin arbiters. A 32-node routing architecture was employed for the latency analysis. In this data collection setup, each dummy processor sequentially sends traffic data to the subsequent node as its destination. Both experiments were conducted on the same setup, and data collections were carried out in an identical architecture, with the only variable being the arbitration techniques employed.

**Figure 13:**
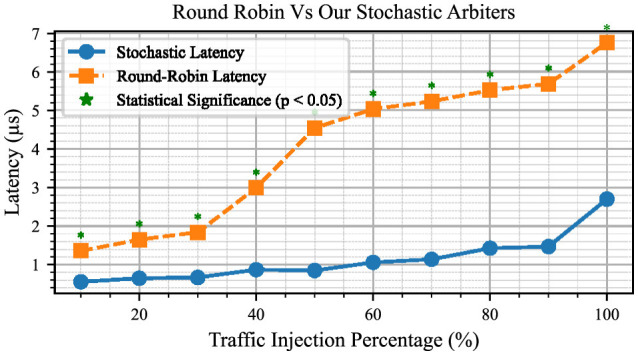
Latency measurements for the design using round-robin arbiters vs. stochastic arbiters.

Figure 13 presents a comparison of the latency between the routing architecture with the round-robin arbitration technique and our stochastic arbitration technique, with both data sets plotted on the same graph. By examining this combined graph, we can analyse and compare the results to better understand the worst-case latency when all nodes inject traffic into the system. It is evident that the stochastic process has less than half the latency of the round-robin arbitration technique. To provide a robust statistical comparison, we conducted a series of *t*-tests at each traffic injection rate to assess the significance of the observed differences between the two arbitration techniques. The *p*-values resulting from these tests were evaluated, and data points where the stochastic arbitration outperformed the round-robin arbitration with statistical significance (*p* < 0.05) are marked on the graph with an asterisk (*). This analysis highlights the consistent latency reduction achieved by the stochastic arbitration technique while validating that the observed improvements are statistically significant.

### 4.5 Resource analysis

This section delves into the resource analysis for various process nodes and routing configurations within the loopback prototype design implemented on the Arria 10 SoC FPGA. The analysis includes resource utilization metrics for systems both with and without dummy processor computing cores, as well as a comparison of logic size and complexity between the Round-Robin and Stochastic arbiters (Tables 1–3). Specifically, Table 1 presents the resource utilization on the Arria 10 SoC FPGA, including processors, for different node configurations. Table 2 provides the resource utilization excluding processors, and Table 3 compares the logic utilization and total registers between the Round-Robin and Stochastic arbiters.

**Table 1:** Resource utilization on Arria 10 SoC FPGA (including processors).

**Node configuration**	**Logic utilization (ALMs) 251,680**	**Total block memory bits 43,642,880**
16 nodes	50,558 (12%)	3,026,944 (7%)
32 nodes	96,790 (22%)	3,387,392 (8%)
64 nodes	189,335 (44%)	4,108,288 (9%)

**Table 2:** Resource utilization on Arria 10 SoC FPGA (excluding processors).

**Node configuration**	**Logic utilization (ALMs) 251,680**	**Total block memory bits 43,642,880**
16 nodes	12,928 (5%)	3,043,328 (7%)
32 nodes	17,817 (7%)	3,403,776 (8%)
64 nodes	27,667 (11%)	4,124,672 (9%)

**Table 3:** Logic utilization and total registers comparison between round robin and stochastic arbiters.

	**Round robin arbiter**	**Stochastic arbiter**
Logic utilization (ALMs)	47/251,680 (< 1%)	182/251,680 (< 1%)
Total registers	13	53

## 5 Discussion

Our motivation for this work is to develop a scalable design that achieves low-latency communication, which is crucial for effective inter-FPGA communication in brain-scale simulations. A scalable design ensures that the system can handle the growing complexity and volume of data, while minimizing delays in data transfer is essential for managing the extensive interactions required in such simulations. To this end, our arbitration technique is designed to minimize buffer congestion by considering the buffer depth at each clock cycle. This approach directly reduces latency. According to authors' knowledge, Astrobyte and SNAVA do not deploy any specific strategies to handle congestion on their designs; the work presented in Bluehive aims to keep the network lightly loaded to reduce the risk of congestion and achieve low-latency routing based on that strategy. However, it is difficult to find sufficient information to compare latency and throughput analysis with Bluehive and SNAVA, as there is limited data available.

In summary, this paper details the multi-FPGA architecture proposed in our work. The proposed architecture employs a NoC-based hierarchical routing approach with a unique arbitration technique. The design covers several aspects. These include router structure, routing logic, data transfer protocol, and arbitration technique. It also focuses on inter-FPGA data communication methods and computing cores used within the design's nodes. This paper evaluates the performance of the multi-FPGA platform. A sample prototype design using two Arria 10 FPGAs and two Stratix V FPGAs illustrates the hierarchical-level communication and scalable routing platform. The prototype design is evaluated with relevant metrics and data. The data from our four-FPGA non loopback prototype design has been compared with a loopback prototype design implemented on Arria 10 SoC FPGA. We explain the experimental setups used for performance calculations, showcasing figures on latency. According to the latency performance, our architecture guarantees low latency for various experimental setups.

The work we have presented here compares a routing architecture using round-robin arbiters by replacing our proposed stochastic arbiters with round-robin arbiters. When comparing the latency performance of two routing architectures under maximum load, our proposed stochastic arbiter-based work shows a notable improvement over the round-robin system. The worst-case latency for a system utilizing round-robin arbiters reaches up to ~6.75 × 10^−6^ s when all nodes are sending traffic at 100% capacity. On the other hand, our system exhibits a worst-case latency of around 2.7 × 10^−6^ s under the same conditions. Hence, our proposed system reduces the worst-case latency by about 60% compared to the system that utilizes round-robin arbiters, highlighting its efficiency in managing high-traffic loads. Additionally, a comparison of design specifications is provided for the proposed system and three other approaches: AstroByte (Karim et al., 2020), SNAVA (Sripad et al., 2018), and Bluehive (Moore et al., 2012). These multi-FPGA platforms are designed for SNN simulations and offer varying levels of programmability. Since these systems were implemented with a diverse range of approaches, each has its advantages and disadvantages. The comparison of specifications is shown in Table 4.

**Table 4:** Comparison between Bluehive, SNAVA and AstroByte.

	**Bluehive**	**SNAVA**	**AstroByte**	**This work**
Topology	3D Torus	Ring	Mesh	Hierarchical Tree
Boards/nodes	16	2	4	128*
Arbitration	N/A	N/A	Round-robin	Stochastic approach-based arbitration tequenique
Routing scheme	Dimension-ordered	New	XY	Dimension-ordered
HDL language	BSV- Bluespec System Verilog	N/A	VHDL	Verilog
Board to board bandwidth (per link)	6Gbps	N/A	4.8Gbps	10.3Gbps
Interconnect method	SATA cable	Ethernet cable	SATA cable	Optical fiber cable
FPGA-to-FPGA mean latency	10 clk cycles	N/A	N/A	13 clk cycles

^*^The proposed architecture consists of 128 nodes. A system with up to 104 nodes on the prototype loopback system has been implemented and analyzed in our work.

The primary innovation of the proposed architecture, aimed at achieving low latency, resides in a stochastic process-based arbitration technique. This technique monitors the number of awaiting queues in each input-output FIFO at every clock cycle, effectively controlling FIFO reading and thereby enhancing latency. Through the combination of the arbitrator technique and the hierarchical routing flexibility, the system demonstrates significant improvements in latency performance.

## Data Availability

The raw data supporting the conclusions of this article will be made available by the authors, without undue reservation.
